# Women Are at a Higher Risk of Chronic Metabolic Diseases Compared to Men With Increasing Body Mass Index in China

**DOI:** 10.3389/fendo.2020.00127

**Published:** 2020-03-12

**Authors:** Xiao-He Wang, Jing-Na Lin, Guang-Zhong Liu, Hai-Ming Fan, Ya-Ping Huang, Chun-Jun Li, Hong-Yuan Yan

**Affiliations:** ^1^College of Public Health, Hebei University, Baoding, China; ^2^Department of Endocrinology, Health Management Center, Tianjin Union Medical Center, Nankai University Affiliated Hospital, Tianjin, China; ^3^Tianjin Municipal Health Commission, Tianjin, China

**Keywords:** obesity, body mass index, chronic non-communicable disease, metabolic disease, gender difference, primary prevention, Chinese adults

## Abstract

**Background:** Chronic non-communicable diseases are the major causes of mortality in the world. However, few studies have investigated the association between multi-categories BMI and chronic diseases from perspective of sex stratification. This study aimed to investigate the risk of chronic diseases at different BMI levels, and to further explore whether BMI-health risk associations differ by sex.

**Methods:** In total, 21,134 participants aged 19–65 years (60.4% men) from the Tianjin People's Hospital, Tianjin Union Medical Center-Health Management Center were recruited for this cross-sectional study. Sex-specific percentiles of BMI were calculated and divided into 11 categories according to the 2000 CDC growth charts. Health-related indicators, such as hyperglycemia, hypertension, non-alcoholic fatty liver diseases (NAFLD), hyperuricemia, etc., were used as dependent variables in this study. Statistical differences were tested by unpaired Mann–Whitney U-test and chi-squared test. Logistic regression models were used to examine the associations between BMI and health-related indicators.

**Results:** The risk of hyperglycemia (OR: 1.67, 95%CI: 1.23–2.29), NAFLD (OR: 2.22, 95%CI: 1.74–2.85), hypertriglyceridemia (OR: 1.65, 95%CI: 1.28–2.12), and hyperuricemia (OR: 1.39, 95%CI: 1.12–1.72) in men began to increase significantly when BMI was in the range of 22.59–23.89 kg/m^2^. However, in women, the risk of hyperglycemia (OR: 3.02, 95%CI: 1.25-8.98) and hyperuricemia (OR: 1.94, 95%CI: 1.26–3.05) began to increase significantly when BMI was in the range of 22.76–23.62 kg/m^2^, and the risk of NAFLD (OR: 5.48, 95%CI: 2.49–14.47) began to increase significantly when BMI was in the range of 21.08–21.97 kg/m^2^. Besides, at the same BMI level, the risk of diseases in women were significantly higher than that in men, especially when BMI > 25 kg/m^2^.

**Conclusion:** In the Chinese population, the risk of chronic diseases in women were significantly higher than that in men at the same BMI level, especially when BMI was >25 kg/m^2^. In addition, the risk of chronic diseases began to increase significantly when BMI was >21.97 kg/m^2^ in women and 23.89 kg/m^2^ in men. The results indicated that women should be more alert to the risk of chronic diseases caused by the increase of BMI than men.

## Introduction

As a global public health issue, chronic non-communicable diseases (NCDs), including hypertension, diabetes, hypercholesterolemia, cardiovascular diseases etc., are the major causes of mortality in the world, especially in developing countries. Until 2016, there were more than 41 million deaths from NCDs in the world, which was 71.3% of total deaths (1). In China, the prevalence of chronic diseases also deserved attention as economic levels increased. For example, according to the data in recent years, the prevalence of diabetes, hypertension (2), hyperuricemia (3), and fatty liver (4) were 11.4% (12.8% for men, 10.6% for women), 37.3% (39.1% for men, 36.2% for women), 13.3% (19.4% for men, 7.9% for women), and 24.6% (31.8% for men, 12.9% for women), respectively. Obesity, as a common risk factor for NCDs, has been shown to be closely associated with various diseases (5–7) and health-related biochemical indicators (8–10). Furthermore, a large number of studies have explored the predictive value of body mass index (BMI) for the risk of chronic diseases by categorizing the population into normal, overweight, and obesity. (11, 12) Obesity control can effectively improves the health of individuals. Therefore, identifying high-risk groups in the early stage of disease or even in the absence of disease, and providing appropriate interventions (13) are considered as the best strategy to save disease prevention costs and to effectively control the disease risks.

However, the existence of sex differences seems to be ignored when studying the association between obesity and chronic diseases. For example, a systematic review found that women with diabetes had a significantly higher risk of coronary heart disease, cardiovascular disease (CVD) mortality and all-cause mortality than men (14). Considering the sex differences (15), the health risks of the population cannot be adequately predicted by dividing the population into 4 categories: underweight, normal, overweight and obesity (16). As a main indicator of obesity, BMI can be divided into more categories for the prediction of health risks of men and women independently. For example, a population-based follow-up cohort study conducted by Rolandsson et al. showed that BMI measured at baseline is regarded as effective as 2 h plasma glucose and fasting plasma glucose for predicting diabetes in Swedish adults. (17) According to a prospective study conducted by Tirosh et al. on 37,674 apparently healthy Israeli men (18) showed that increased BMI in childhood and adolescence might be closely associated with higher incidences of coronary heart disease and type 2 diabetes mellitus in young adults. In addition, a systematic review conducted by Zaccardi et al. (19) studied the association between BMI and all-cause and cardiovascular mortalities in type 2 diabetes mellitus patients, which showed a strong non-linear association between BMI and all-cause mortality, with the lowest estimated risk from ranges from 31 to 35 kg/m^2^ for men and 28–31 kg/m^2^ for women, respectively. Although these studies have involved multi-categories BMI (i.e., more than four groups), they were all based on the Western populations and did not consider sex differences. Studies regarding the association between sex-specific multi-categories BMI and health risks in Chinese are still limited (20–22).

Due to lack of studies on the association between multi-categories BMI and health risks in China, we conducted a population-based cross-sectional study to investigate the risk of chronic diseases at different BMI levels, and to further explore whether BMI-health risk associations differ by sex.

## Materials and Methods

### Participants

A total of 38,452 participants for annual health examination from the Tianjin Union Medical Center-Health Management Center, which is mainly based on group check-up, during Jan 2016 to Dec 2018 were enrolled. In order to ensure the representativeness of the study subjects, those who participated in the health examination for annual routine physical examination were included in the study. Participants who did not provide complete information, such as height, weight, fasting plasma glucose, blood pressure, B-ultrasound, and other relevant biochemical indicators (*n* = 14,676), and those with a history of cancer (*n* = 164) were excluded. We also excluded participants below 18 years of age and above 65 (*n* = 2,457), as the association between BMI and chronic diseases due to stability of physiological indicators or serious declination of health status might be affected. Furthermore, for those who have repeated measurement data (*n* = 21), the most recent data was selected and excluded the previous. Finally, a total of 21,134 subjects with physical examination data were included in this study. This study was approved by the Institutional Review Board of Tianjin Union Medical Center, Nankai University affiliated hospital. Participants were informed about the study objectives and examination procedures in detailed, and were asked to sign the informed consent form before participating in this study.

### Procedures

Weight and height were measured by an automatic height and weight instrument (DST-600, DONGHUAYUAN, China), and participants were required to be on barefoot and light clothing during the measurement. BMI was calculated as weight in kilograms divided by the square of height in meters. Blood pressure was measured using an automatic electronic blood pressure monitor (AC-05C, Ling Qian, China) after a 10 min rest, and measured thrice per person and averaged. The history of diseases, including diabetes, high blood pressure, cancer and coronary heart disease (CHD), were obtained through self-reporting. The diagnosis of NAFLD was performed by ultrasound diagnosis system (Phoenix, Philips and Neusoft Medical Systems Co., Ltd., China). Blood biochemical analysis was performed using an automatic biochemical analyzer (TBA-120FR, Toshiba, Japan), and participants were required to fast overnight (only water can be taken), and the venous blood was collected in a fasting state. The main indicators of blood biochemical analysis include fasting plasma glucose (FPG), total cholesterol (TC), triglycerides (TG), low-density lipoprotein (LDL-C) and high-density lipoprotein (HDL-C) cholesterol, and uric acid. All measurements are carried out in strict accordance with the national standards.

### Outcome Variables

In order to study the association between multi-categories BMI and health risks, we selected 8 health-related indicators such as hyperglycemia, hypertension, NAFLD, hypercholesterolemia, hypertriglyceridemia, LDL-C hyperlipidemia, HDL-C hypolipidemia and hyperuricemia. The prevalence of the diseases was used as dependent variables, and the sex-specific percentiles of BMI were used as independent variables. Hyperglycemia was defined as FPG ≥ 7.0 or the subjects with a history of diabetes. Dyslipidemia were defined as TC ≥ 6.2 mmol/L or TG ≥ 2.3 mmol/L or LDL-C ≥ 4.1 mmol/L or HDL-C <1.0 mmol/L according to the Chinese guidelines for the management of dyslipidemia in adults (2016) (23). Hypertension is defined as systolic blood pressure ≥ 140 mmHg and/or diastolic ≥ 90 mmHg or the subjects who had a history of hypertension. Hyperuricemia is defined as a serum uric acid level ≥ 416 μmol/L in men and menopausal women, and ≥ 357 μmol/L in premenopausal women. (24) NAFLD, including mild fatty liver, moderate fatty liver and severe fatty liver were directly diagnosed by B-ultrasound. BMI percentiles were calculated and divided into 11 categories according to the 2000 CDC growth charts by sex (25). In addition, the BMI was divided into 4 categories according to the Chinese Obesity Standard (26) for comparison.

### Statistical Analysis

Given that the distributions of the continuous variables in this study was not normal, the characteristics of participants were presented as median with interquartile range (IQR) for continuous variables and frequencies for categorical variables. Statistical significance between men and women was tested for continuous and categorical variables by unpaired Mann–Whitney U-test and chi-squared test, respectively. BMI was divided into 11 categories by percentiles (the second interval was taken as the reference level) and the percentiles of men and women were calculated separately. The association between multi-categories BMI and health-related indicators were assessed by logistic regression analysis to estimate the Odds ratios (ORs) and 95% confidence intervals (CIs). The *P*-values for linear trends were calculated using the median value of each intervals of BMI. All statistical analyses were performed with Statistical Analysis System 9.4 edition for Windows (SAS Institute, Cary, NC, USA). *P*-values were two-tailed, and the differences were considered to be significant when *P* < 0.05.

## Results

**Figure 1:**
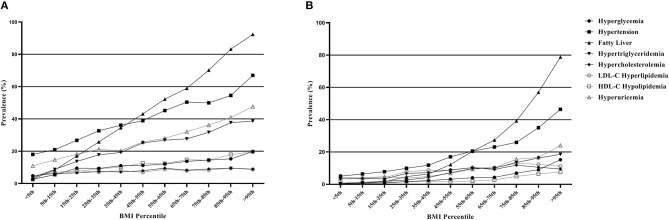
**(A)** Prevalence of metabolic diseases in men according to body mass index (BMI) percentiles. **(B)** Prevalence of metabolic diseases in women according to body mass index (BMI) percentiles. The BMI percentile is calculated separately for men and women.

The characteristics of participant according to sex are presented in Table 1. There are significant differences between men and women (*P* < 0.0001) in almost all indicators, except for hypercholesterolemia (*P* = 0.9852). The median of BMI (26.12 kg/m^2^) in men was significantly higher than that of women (23.15 kg/m^2^) and was at an overweight level. Besides, in addition to LDL-C hyperlipidemia, the prevalence of the diseases studied in men was more than twice that in women. Figure 1 shows the prevalence of diseases after BMI percentile stratification. It can be seen that the prevalence of the diseases studied is rising rapidly with the increase of BMI, especially in NAFLD and hypertension. The prevalence of the two diseases crossed over in the 35th to 45th percentiles (BMI range: 24.82–25.68 kg/m^2^) for men and 55th to 65th percentiles (BMI range: 23.62–24.61 kg/m^2^) for women, respectively.

**Table 1:** Characteristics of the study population according to sex^a^.

	**Total (*n =* 21,134)**	**Men (*n =* 12,769)**	**Women (*n =* 8,365)**	***P-Value*^b^**
Age, y	44 (35, 54)	44 (36, 55)	43 (34, 52)	<0.0001
Weight, kg	71 (61, 81)	78 (70, 86)	60 (54, 67)	<0.0001
Height, kg	168 (162, 174)	173 (169, 177)	161 (157, 165)	<0.0001
BMI, kg/m^2^	25.01 (22.48, 27.76)	26.12 (23.89, 28.69)	23.15 (21.08, 25.78)	<0.0001
SBP, mmHg	123 (112, 135)	127 (117, 139)	116 (105, 128)	<0.0001
DBP, mmHg	81 (74, 89)	84 (77, 91)	77 (70, 84)	<0.0001
FPG, mmol/L	5.2 (4.86, 5.64)	5.31 (4.96, 5.83)	5.04 (4.73, 5.39)	<0.0001
TG, mmol/L	1.28 (0.88, 1.92)	1.5 (1.05, 2.21)	0.99 (0.71, 1.44)	<0.0001
TC, mmol/L	4.75 (4.19, 5.39)	4.79 (4.24, 5.42)	4.69 (4.14, 5.34)	<0.0001
LDL-C, mmol/L	3.09 (2.64, 3.55)	3.1 (2.65, 3.56)	3.06 (2.62, 3.53)	0.0358
HDL-C, mmol/L	1.3 (1.14, 1.5)	1.22 (1.09, 1.39)	1.43 (1.25, 1.64)	<0.0001
Uric acid, umol/L	325 (266, 390)	367 (316, 422)	264 (227, 307)	<0.0001
Hyperglycemia (%)	8.47	11.34	4.09	<0.0001
Hypertension (%)	31.33	39.81	18.39	<0.0001
Non-alcoholic fatty liver (%)	34.99	44.15	21	<0.0001
Hypercholesterolemia (%)	7.85	7.85	7.85	0.9852
Hypertriglyceridemia (%)	16.92	22.99	7.65	<0.0001
LDL-C Hyperlipidemia (%)	8.17	7.82	8.71	0.0196
HDL-C Hypolipidemia (%)	8.09	11.57	2.77	<0.0001
Hyperuricemia (%)	19.68	26.67	9.01	<0.0001
History of diseases (%)				
Diabetes	3.62	4.97	1.57	<0.0001
Hypertension	7.22	9.19	4.21	<0.0001
CHD	1.29	1.67	0.71	<0.0001

*BMI, body mass index; SBP, systolic blood pressure; DBP, diastolic blood pressure; FPG, fasting plasma glucose; TG, triglycerides; TC, total cholesterol; LDL-C, low-density lipoprotein cholesterol; HDL-C, high-density lipoprotein cholesterol; CHD, coronary heart disease*.

a *Continuous variable is expressed as median with interquartile range and categorical variables are expressed as frequencies*.

b *Unpaired Mann–Whitney U-test or chi-squared test was performed where appropriate*.

The association between sex-specific percentiles of BMI and health-related indicators was presented in Table 2. Among men, the risk of hyperglycemia (OR: 1.67, 95% CI: 1.23–2.29), hypertension (OR: 1.29, 95% CI: 1.07–1.56), NAFLD (OR: 2.22, 95% CI: 1.74–2.85), hypertriglyceridemia (OR: 1.65, 95% CI: 1.28–2.12), HDL-C hypolipidemia (OR: 1.44, 95% CI: 1.05–1.98), and hyperuricemia (OR: 1.39, 95% CI: 1.12–1.72) began to increase significantly in the 15th to 25th percentiles (BMI range: 22.59–23.89 kg/m^2^), and the risk of LDL-C hyperlipidemia (OR: 1.48, 95% CI: 1.11–1.98) began to increase significantly in the 85th to 95th percentiles (BMI range: 30.09–33.08 kg/m^2^). Among women, the risk of NAFLD (OR: 5.48, 95% CI: 2.49–14.47) began to increase significantly in the 25th to 35th percentiles (BMI range: 21.08–21.97 kg/m^2^). The risk of hypertriglyceridemia (OR: 2.83, 95% CI: 1.44–6.09) and HDL-C hypolipidemia (OR: 2.91, 95% CI: 1.16–8.26) began to increase significantly in the 35th to 45th percentiles (BMI range: 21.97–22.76 kg/m^2^). The risk of hyperglycemia (OR: 3.02, 95% CI: 1.25–8.98), hypertension (OR: 1.79, 95% CI: 1.28–2.54) and hyperuricemia (OR: 1.94, 95% CI: 1.26–3.05) began to increase significantly in the 45th to 55th percentiles (BMI range: 22.76–23.62 kg/m^2^), and the risk of LDL-C hyperlipidemia (OR: 1.89, 95% CI: 1.26–2.93) began to increase in the 75th to 85th percentiles (BMI range: 25.78–27.34 kg/m^2^). For comparison, the association between traditional four-categories BMI and health-related indicators were showed in Table 3. Unlike multi-categories BMI, no significant sex differences were observed in the traditional four-categories BMI.

**Table 2:** Age- and history of diseases-adjusted odds ratios with 95% confidence intervals for the association of sex-specific percentiles of BMI and health-related indicators stratified by sex^a^.

**Health-related indicators**	**Percentiles of BMI**											**P for Trend^b^**
	**<5th**	**5th−15th**	**15th−25th**	**25th−35th**	**35th−45th**	**45th−55th**	**55th−65th**	**65th−75th**	**75th−85th**	**85th−95th**	**>95th**	
**MEN**												
Num. of cases	632	1,266	1,294	1,277	1,274	1,260	1,295	1,262	1,288	1,279	642	
Hyperglycemia	0.57 (0.32, 0.96)	1.00 (ref)	1.67 (1.23, 2.29)	1.67 (1.22, 2.29)	1.99 (1.47, 2.72)	2.07 (1.53, 2.82)	2.27 (1.68, 3.08)	2.70 (2.02, 3.66)	3.12 (2.34, 4.22)	3.84 (2.87, 5.18)	6.80 (4.94, 9.45)	<0.0001
Hypertension	0.95 (0.74, 1.22)	1.00 (ref)	1.29 (1.07, 1.56)	1.76 (1.46, 2.12)	2.03 (1.69, 2.43)	2.32 (1.94, 2.79)	3.05 (2.55, 3.65)	3.84 (3.21, 4.60)	3.97 (3.32, 4.75)	5.28 (4.42, 6.33)	10.57 (8.49, 13.20)	<0.0001
Non-alcoholic fatty liver	0.33 (0.19, 0.53)	1.00 (ref)	2.22 (1.74, 2.85)	3.77 (2.99, 4.79)	5.70 (4.55, 7.20)	8.22 (6.57, 10.37)	11.89 (9.51, 14.98)	15.60 (12.46, 19.69)	25.70 (20.47, 32.56)	54.52 (42.76, 70.13)	135.18 (95.86, 194.46)	<0.0001
Hypercholesterolemia	0.83 (0.53, 1.26)	1.00 (ref)	1.02 (0.74, 1.40)	1.17 (0.86, 1.59)	1.11 (0.81, 1.51)	1.25 (0.92, 1.70)	1.52 (1.13, 2.04)	1.30 (0.96, 1.77)	1.47 (1.09, 1.99)	1.62 (1.21, 2.19)	1.62 (1.13, 2.30)	<0.0001
Hypertriglyceridemia	0.40 (0.24, 0.61)	1.00 (ref)	1.65 (1.28, 2.12)	2.25 (1.77, 2.87)	2.47 (1.95, 3.15)	3.49 (2.77, 4.42)	3.81 (3.04, 4.82)	3.97 (3.16, 5.02)	4.82 (3.85, 6.07)	6.30 (5.05, 7.92)	6.58 (5.13, 8.49)	<0.0001
LDL-C Hyperlipidemia	0.73 (0.47, 1.11)	1.00 (ref)	1.12 (0.83, 1.51)	0.98 (0.72, 1.33)	1.11 (0.82, 1.50)	0.97 (0.71, 1.32)	1.25 (0.94, 1.68)	1.13 (0.84, 1.53)	1.21 (0.90, 1.63)	1.48 (1.11, 1.98)	1.45 (1.02, 2.06)	<0.0001
HDL-C Hypolipidemia	0.42 (0.24, 0.72)	1.00 (ref)	1.44 (1.05, 1.98)	1.75 (1.29, 2.39)	2.01 (1.49, 2.73)	2.55 (1.91, 3.44)	2.51 (1.88, 3.38)	3.05 (2.30, 4.09)	2.93 (2.21, 3.93)	3.72 (2.82, 4.95)	3.98 (2.92, 5.45)	<0.0001
Hyperuricemia	0.63 (0.47, 0.85)	1.00 (ref)	1.39 (1.12, 1.72)	1.68 (1.37, 2.07)	1.63 (1.32, 2.01)	2.22 (1.81, 2.73)	2.52 (2.07, 3.09)	3.05 (2.50, 3.72)	3.55 (2.92, 4.32)	4.08 (3.36, 4.96)	4.88 (3.91, 6.10)	<0.0001
**WOMEN**												
Num. of cases	413	830	805	877	827	846	836	835	835	841	420	
Hyperglycemia	0.96 (0.14, 4.51)	1.00 (ref)	1.04 (0.31, 3.64)	2.02 (0.77, 6.26)	2.44 (0.97, 7.44)	3.02 (1.25, 8.98)	3.70 (1.56, 10.9)	3.67 (1.55, 10.79)	6.33 (2.76, 18.32)	8.82 (3.89, 25.34)	19.61 (8.54, 56.76)	<0.0001
Hypertension	0.94 (0.54, 1.59)	1.00 (ref)	1.08 (0.73, 1.60)	1.18 (0.82, 1.71)	1.31 (0.92, 1.89)	1.79 (1.28, 2.54)	2.20 (1.58, 3.11)	2.43 (1.75, 3.43)	2.93 (2.12, 4.11)	4.87 (3.55, 6.80)	10.14 (7.16, 14.54)	<0.0001
Non-alcoholic fatty liver	0.77 (0.11, 3.38)	1.00 (ref)	2.03 (0.79, 5.80)	5.48 (2.49, 14.47)	6.97 (3.21, 18.25)	12.95 (6.14, 33.39)	23.86 (11.43, 61.10)	34.21 (16.46, 87.42)	61.42 (29.65, 156.62)	135.11 (65.28, 344.33)	471.33 (221.41, 999.99)	<0.0001
Hypercholesterolemia	1.14 (0.57, 2.18)	1.00 (ref)	0.77 (0.44, 1.32)	1.35 (0.86, 2.17)	1.30 (0.83, 2.09)	1.44 (0.93, 2.28)	1.59 (1.04, 2.51)	1.30 (0.84, 2.06)	1.79 (1.17, 2.81)	1.61 (1.05, 2.54)	1.66 (1.00, 2.77)	0.0007
Hypertriglyceridemia	0.45 (0.07, 1.72)	1.00 (ref)	1.43 (0.65, 3.32)	1.90 (0.93, 4.20)	2.83 (1.44, 6.09)	4.61 (2.44, 9.65)	6.22 (3.34, 12.91)	6.25 (3.36, 12.98)	8.88 (4.83, 18.29)	11.25 (6.16, 23.08)	14.56 (7.78, 30.36)	<0.0001
LDL-C Hyperlipidemia	1.32 (0.70, 2.43)	1.00 (ref)	0.89 (0.53, 1.49)	1.51 (0.98, 2.38)	1.51 (0.98, 2.37)	1.30 (0.85, 2.05)	1.52 (0.99, 2.36)	1.37 (0.90, 2.14)	1.89 (1.26, 2.93)	1.83 (1.21, 2.83)	1.89 (1.17, 3.09)	<0.0001
HDL-C Hypolipidemia	1.49 (0.43, 4.97)	1.00 (ref)	2.03 (0.77, 5.91)	2.44 (0.96, 6.98)	2.91 (1.16, 8.26)	3.36 (1.35, 9.50)	3.95 (1.62, 11.06)	5.60 (2.40, 15.32)	10.21 (4.61, 27.09)	13.81 (6.34, 36.26)	14.71 (6.51, 39.46)	<0.0001
Hyperuricemia	1.00 (0.54, 1.8)	1.00 (ref)	1.22 (0.76, 1.98)	0.99 (0.61, 1.63)	1.31 (0.82, 2.11)	1.94 (1.26, 3.05)	2.74 (1.81, 4.23)	3.09 (2.05, 4.75)	4.95 (3.36, 7.50)	5.35 (3.64, 8.09)	8.26 (5.50, 12.70)	<0.0001

a *Values are presented as OR (95% CI) unless otherwise indicated. BMI, body mass index; LDL-C, low-density lipoprotein cholesterol; HDL-C, high-density lipoprotein cholesterol*.

b *P-values for linear trends were calculated using the median value for each category*.

**Table 3:** Age- and history of diseases-adjusted odds ratios with 95% confidence interval for the association between BMI and health-related indicators stratified by sex^a^.

**Health-related indicators**	**BMI**				***P*-Value^b^**
	**<18.5**	**18.5–23.9**	**24.0–27.9**	**> = 28.0**	
**MEN**					
No. of subjects	190	3,147	5,475	3,957	
Hyperglycemia	0.31 (0.08, 0.83)	1.00 (ref.)	1.65 (1.41, 1.95)	2.82 (2.39, 3.34)	<0.0001
Hypertension	0.51 (0.31, 0.8)	1.00 (ref.)	2.09 (1.89, 2.31)	4.33 (3.89, 4.82)	<0.0001
Non-alcoholic fatty liver	0.04 (0, 0.18)	1.00 (ref.)	5.07 (4.5, 5.73)	23.67 (20.8, 26.99)	<0.0001
Hypercholesterolemia	0.44 (0.16, 0.98)	1.00 (ref.)	1.23 (1.03, 1.46)	1.41 (1.18, 1.69)	<0.0001
Hypertriglyceridemia	0.14 (0.03, 0.36)	1.00 (ref.)	2.53 (2.22, 2.89)	4.38 (3.85, 5.01)	<0.0001
LDL-C Hyperlipidemia	0.41 (0.14, 0.91)	1.00 (ref.)	1.06 (0.9, 1.25)	1.28 (1.07, 1.52)	0.0005
HDL-C Hypolipidemia	0.15 (0.03, 0.48)	1.00 (ref.)	2.01 (1.7, 2.39)	3.08 (2.61, 3.66)	<0.0001
Hyperuricemia	0.38 (0.21, 0.62)	1.00 (ref.)	1.77 (1.58, 1.99)	3.3 (2.94, 3.71)	<0.0001
**WOMEN**					
No. of subjects	408	4,522	2,437	998	
Hyperglycemia	0.45 (0.07, 1.46)	1.00 (ref.)	2.44 (1.85, 3.24)	5.72 (4.25, 7.72)	<0.0001
Hypertension	0.72 (0.44, 1.12)	1.00 (ref.)	2.13 (1.86, 2.44)	4.94 (4.18, 5.84)	<0.0001
Non-alcoholic fatty liver	0.11 (0.02, 0.35)	1.00 (ref.)	6.52 (5.61, 7.6)	33.27 (27.64, 40.18)	<0.0001
Hypercholesterolemia	0.97 (0.52, 1.65)	1.00 (ref.)	1.34 (1.12, 1.61)	1.31 (1.01, 1.67)	0.0041
Hypertriglyceridemia	0.17 (0.03, 0.54)	1.00 (ref.)	2.81 (2.31, 3.43)	4.48 (3.57, 5.62)	<0.0001
LDL-C Hyperlipidemia	1.05 (0.6, 1.71)	1.00 (ref.)	1.24 (1.04, 1.48)	1.49 (1.18, 1.88)	0.0004
HDL-C Hypolipidemia	0.67 (0.23, 1.53)	1.00 (ref.)	3.49 (2.52, 4.87)	6.17 (4.33, 8.8)	<0.0001
Hyperuricemia	0.76 (0.44, 1.22)	1.00 (ref.)	2.92 (2.43, 3.5)	4.71 (3.82, 5.8)	<0.0001

a *Values are presented as OR (95% CI) unless otherwise indicated. BMI, body mass index; HDL-C, high-density lipoprotein cholesterol*.

b *P-values for linear trends were calculated using the median value of each intervals of BMI*.

**Figure 2:**
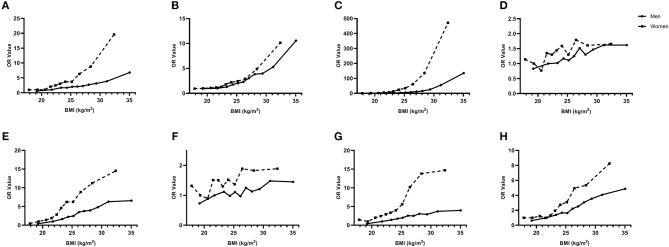
Age-adjusted odds ratio for the association between Body Mass Index (BMI) and health-related indicators. **(A)** Hyperglycemia; **(B)** Hypertension; **(C)** Non-alcoholic fatty liver disease; **(D)** Hypercholesterolemia; **(E)** Hypertriglyceridemia; **(F)** LDL-C Hyperlipidemia; **(G)** HDL-C Hypolipidemia; **(H)** Hyperuricemia. BMI was divided into 11 categories according to percentiles.

In addition, the risk of the diseases was compared between men and women by converting BMI classifications into continuous variables (Figure 2). It can be seen that with the increase of BMI, the risk of diseases has increased significantly both in men and women, except for LDL-C hyperlipidemia and hypercholesterolemia. Besides, at the same BMI level, the risk of diseases in women was significantly higher than that in men, and when BMI > 25 kg/m^2^, the risk of chronic diseases in women significantly increased faster than that in men, a rapid trend separation was observed in hyperglycemia, NAFLD, hypertriglyceridemia, HDL-C hypolipidemia and hyperuricemia.

## Discussion

This is the first study based on a large sample population to investigate the association between BMI and chronic metabolic diseases in Chinese population by dividing BMI into 11 categories (stratified by sex). The results showed that the risk of chronic diseases in women with overweight or obesity was significantly higher than that in men. Besides, the risk of chronic diseases also increased both in men and women when BMI was in the “normal” range (18.5–24 kg/m^2^), and a sharp increase was observed when BMI was in the range of 21.08–21.97 kg/m^2^ in women or 22.59–23.89 kg/m^2^ in men.

Previous studies have shown that women have a higher risk of chronic diseases such as cardiovascular disease and diabetes than men (14, 27). In the present study, it is further found that the risk of diseases in women is significantly higher than men at the same BMI level, and the differences gradually increase when BMI is >25 kg/m^2^, which was consistent with previous study (28). Remarkably, the risk of hyperglycemia, hypertension, NAFLD, hypertriglyceridemia, HDL-C hypolipidemia and hyperuricemia was significantly higher than reference level even within the “normal” level (BMI: 18.5–23.9 kg/m^2^) of traditional BMI classification in men, while in women, a significant increase in the risks of NAFLD and hypertriglyceridemia was observed when the BMI was >21.08 kg/m^2^ and 23.62 kg/m^2^, respectively. There are several possible mechanisms for these results. Firstly, according to the study conducted by Aydin et al. (29) individuals with normal BMI might have a higher body fat, and body fat percentage, especially the visceral fat, and this has been confirmed as a risk factor for chronic diseases (30). This indirectly affected the development of chronic diseases through metabolic risk factors (31). Secondly, the so-called normal BMI range was only a possible interval. The sex-specific percentiles of BMI also included the “normal” range, and we further divided it into smaller intervals. Our results indicated that within these intervals, some of the intervals showed no significant effect on the increase of disease risk. If the proportion of people in these specific intervals increases, it might show a negative impact on the entire range of BMI, resulting in a so-called “normal” BMI range. This might be explained by the study conducted by Tirosh et al. (18) in which the results showed an increased risk of diseases even within the normal BMI range, and obesity in adolescence is probably the only tip of an iceberg of BMI-related increase in the risk of coronary heart disease.

In addition, significant sex differences were observed at higher BMI levels (>25 kg/m^2^) when studying the association between sex-specific percentiles of BMI and health-related indicators. The risk of the indicators, including hyperglycemia, NAFLD, hypertriglyceridemia, low-density lipoproteinemia and hyperuricemia, were significantly higher in women with overweight or obesity than in men. These indicators are mainly related to lipid metabolism and inflammation, suggesting a significant difference in the mechanisms of lipid metabolism and inflammation between men and women. In consistent with the previous studies (32, 33), lipid metabolic disorders are one of the major metabolic changes in population with obesity, and further inflammation plays a key role in increasing health risk (such as type 2 diabetes and NAFLD) in the population. Our results suggested that women with obesity seem to be more susceptible to the adverse health outcomes caused by inflammation and lipid metabolism disorders than men with obesity. Differences in skeletal muscle mass between men and women may partly explain the result. A review conducted by Zhang et al. demonstrated that significant sex differences in glucose and lipid metabolism of skeletal muscle may further affect the risk of type 2 diabetes in men and women (34). In addition to lipid metabolism and inflammation, the difference in the role of sex hormones between men and women may be another potential mechanism. On the one hand, sex hormones can affect the distribution of body fat, and as we know, the increase of visceral fat is a risk factor for various chronic diseases (35); on the other hand, sex hormones can indirectly affect human behavior, such as dietary preference or physical activity level, which may also affect the prevalence of chronic diseases to some extent. A systematic review confirms that hyperandrogenism may increase the risk of type 2 diabetes in women while decreasing risk in men (36). Similarly, Kalyani et al. also confirmed that sex hormones could partially explained the association between sex hormones and type 2 diabetes among postmenopausal women (37). However, it remains to be determined whether obesity further contributes to the association.

Furthermore, significant sex differences were also observed in BMI intervals below the reference level. Lower BMI seems to be a protective factor for the diseases studied in men, while no significant association was observed in women, and further adjustment for age did not change the association. However, the relevant mechanisms have not yet been clarified.

There are two advantages in this study. Firstly, this is a large population-based study, and the data was derived from a large integrated health care system with a wide range of customers that reflects the diverse population of Tianjin, China. It's provided a reliable support for the results and can be well-extended to the general population. Secondly, all measurements are performed in strict accordance with the standard procedures, and all statistical analysis was scientifically designed, ensuring the accuracy of the data. However, there are several limitations that also need to be mentioned. Firstly, as a single-center study and based on the cross-sectional data, the conclusions of the present study have some deficiencies in application to the general population. However, we collected large samples and ensured the uniformity of measurements (such as biochemical analysis and physical examination), which can significantly reduce system errors, and made the results we obtained more robust. Secondly, we were unable to make further multiple comparisons to the model, since we did not collect more covariate information, such as the socio-demographics, lifestyle, and behavioral factors of participants, which might be potential confounders that influence the results. Nevertheless, we adjusted for two important factors (age and history of diseases) that make the results more reliable. Finally, given that the routine physical examination items did not include waist circumference and body composition measurement, we could not obtain relevant data. Considering waist circumference (38, 39) and body composition (40, 41) as important supplementary indicators of BMI, they were also significantly related to the prevalence of some chronic diseases. The lack of these information might make us unable to conduct further analysis, and the interpretation of the results is slightly inadequate. Despite these limitations, our results provided valuable information to practically explore the aim of our study. This purpose of this study was to find a simple and practical method to predict high-risk groups of chronic diseases in Chinese population, and therefore our findings could be particularly relevant from a public health point of view.

In conclusion, the present study based on the Chinese population showed that the risk of chronic diseases in women were significantly higher than that in men at the same BMI level, especially when BMI was >25 kg/m^2^. In addition, the risk of chronic diseases began to increase significantly when BMI was >21.97 kg/m^2^ in women and 23.89 kg/m^2^ in men. The results indicated that women should be more alert to the risk of chronic diseases caused by the increase of BMI than men. However, well-designed prospective cohort studies are needed to further confirm our findings and clarify the mechanisms.

## Data Availability Statement

All datasets generated for this study are included in the article/supplementary material.

## Ethics Statement

The studies involving human participants were reviewed and approved by Institutional Review Board of Tianjin Union Medical Center, Nankai University affiliated hospital. The patients/participants provided their written informed consent to participate in this study.

## Author Contributions

C-JL and HY contributed to the concept and design of the study. X-HW and J-NL contributed to the design of analysis, statistical analysis, and manuscript preparation. X-HW, G-ZL, H-MF, and Y-PH contributed to coordination of the fieldwork, data collection and management, interpretation of results, and revision of manuscript drafts. All authors were involved in the interpretation of the results, the revision of the manuscript, and approved the submitted version of the manuscript.

### Conflict of Interest

The authors declare that the research was conducted in the absence of any commercial or financial relationships that could be construed as a potential conflict of interest.

## Abbreviations

NCDs: chronic non-communicable diseases
BMI: body mass index
NAFLD: non-alcoholic fatty liver diseases
CVD: cardiovascular disease
CHD: coronary heart disease
TC: total cholesterol
TG: triglycerides
LDL-C: low-density lipoprotein cholesterol
HDL-C: high-density lipoprotein cholesterol
FPG: fasting plasma glucose
IQR: interquartile range
OR: Odds ratio
CI: confidence intervals.
